# Intuitionistic fuzzy-based model for failure detection

**DOI:** 10.1186/s40064-016-3446-0

**Published:** 2016-11-09

**Authors:** Daniel O. Aikhuele, Faiz B. M. Turan

**Affiliations:** Faculty of Manufacturing Engineering, Universiti Malaysia Pahang, 26600 Pekan, Malaysia

**Keywords:** Exponential related function, Intuitionistic fuzzy entropy, Intuitionistic fuzzy TOPSIS, Failure detection, Product redesign

## Abstract

In identifying to-be-improved product component(s), the customer/user requirements which are mainly considered, and achieved through customer surveys using the quality function deployment (QFD) tool, often fail to guarantee or cover aspects of the product reliability. Even when they do, there are always many misunderstandings. To improve the product reliability and quality during product redesigning phase and to create that novel product(s) for the customers, the failure information of the existing product, and its component(s) should ordinarily be analyzed and converted to appropriate design knowledge for the design engineer. In this paper, a new intuitionistic fuzzy multi-criteria decision-making method has been proposed. The new approach which is based on an intuitionistic fuzzy TOPSIS model uses an exponential-related function for the computation of the separation measures from the intuitionistic fuzzy positive ideal solution (IFPIS) and intuitionistic fuzzy negative ideal solution (IFNIS) of alternatives. The proposed method has been applied to two practical case studies, and the result from the different cases has been compared with some similar computational approaches in the literature.

## Background

The design of most complex products and systems like crane vessel, diesel engines etc., are mainly done by redesigning or making changes on existing predecessor designs until all new arising requirements are met (Smith et al. 2012; Romli and Harmin 2015). The main goal of any product redesigning exercise is to create new products and systems that meet the customer requirements as well as the product reliability. In identifying product component(s) to be redesign, the customer/user requirements which are mainly considered (Risdiyono and Koomsap 2013; Liu et al. 2012, 2014; Shieh et al. 2008; Shin et al. 2015) and achieved through customer surveys using the quality function deployment (QFD) tool, often fail to guarantee or cover aspects of the product reliability. According to Dietrich (2006), only very few customers will specify the traditional reliability requirements in terms of mean time between failures (MTBF), Failure rate or the probability of failure occurrence. Even when they do, there are always many misunderstandings.

To improve the product reliability and quality during the product redesigning phase, and to create that novel product(s) for the customers, deliberate effort must be made to identify and analyze the failure information of the existing product (Yang et al. 2011; Smith et al. 2012) by consulting the historical failure data of the product, where the analyzed information will be used for building appropriate design knowledge for the design engineer(s). The identification of the failed product component is most critical to achieving an improved product quality (He et al. 2015).

The method most commonly used for identifying and analyzing failures during the prototype testing stage is the failure mode and effect analysis (FMEA) method. The FMEA method which was introduced in the 1960s by the United States aerospace industry as a structured and systematic method with apparent reliability and safety requirements (Bowles and Pelaez 1995) has proven to be a popular engineering technique for identifying, ranking and evaluating potential failures in new and existing products as well as in the improvement of product quality.

The FMEA method is an analytical tool that can be used to ensure the safety and reliability of both new and existing products, it allows for an objective approach in making risk-based management decisions in a wide range of industries including the aerospace industry, automotive, nuclear, healthcare and the shipping industry etc. (Chang et al. 2012; Vinodh et al. 2012; Helvacioglu and Ozen 2014; Liu et al. 2013; Hu-Chen et al. 2013; Sayareh and Ahouei 2013). In implementing the FMEA method, a cross-functional team with expertise from different departments in a company are rottenly involved in the systematic evaluation and quantification of the relationships between the failure modes, effects, causes and controls, and to proposed corrective actions for the product (Zhao et al. 2016).

In spite of the fact that the FMEA method is a well-established method for product reliability assessment, however, there are some drawbacks that have been reported with its applications, including the difficulty to accurately and precisely determine the probability of failure event in products (Mohammadi and Tavakolan 2013; Xie 2013). The ineffectiveness of the method to accurately reflect, model or account for design errors, human factors implications, flawed requirements and component interaction accidents in products (Keizer et al. 2005; Liu et al. 2014; Martínez 2015) and finally, the fuzziness and hesitation of the experts’ subjective assessments which are not accounted for, modeled or reflexed in the FMEA technique (Zhao et al. 2016).

In an attempt to solve these problems, several alternative methods and approaches have been presented in the literature. Among them we can mention, the new Euclidean distance-based similarity measure and an incremental learning clustering model presented by Tay et al. (2015), which they applied for clustering failure modes in FMEA in an edible birds nest industry. The fuzzy evidential reasoning and belief rule-based methodology presented by Liu et al. (2013) for prioritizing failures in FMEA. The fuzzy evidential reasoning and grey theory method by Liu et al. (2011).

Geum et al. (2011), presents the service-specific FMEA and grey relational analysis approach for diagnosing service failure, while Netto et al. (2013), presents a mathematical model which is based on data envelopment analysis for analyzing the operational risk of flexible subsea risers and pipelines used for the transportation of oil and gas products.

A number of multi-criteria decision-making (MCDM) methods have also been presented as alternative methods for the FMEA, among them, we can mention; the VlseKriterijumska Optimizacija I Kompromisno Resenje (VIKOR) presented by Safari et al. (2016), for prioritizing enterprise architecture (EA) risk factors. Adhikary et al. (2014), presents an integrated approach which is based on grey number and complex proportional assessment (COPRAS-G). Balin et al. (2014) presents a fuzzy analytic hierarchy process and VIKOR model for failure detection in a marine diesel engine. While Liu et al. (2015), presented an intuitionistic fuzzy hybrid technique for order preference by similarity to ideal solution (TOPSIS) using an intuitionistic fuzzy hybrid weighted Euclidean distance (IFHWED) operator.

In a similar vein, this study is, therefore, presenting a new approach, an intuitionistic fuzzy TOPSIS model which is based on an exponentially related function (IF-TOPSIS_EF_) as an alternative approach for the FMEA method. In this case, the new approach is used for building appropriate design knowledge about the to-be-improved or redesigned product component by analyzing the failure information about the product with the view to prioritizing the failure modes and to provide the designer with relevant information to be used in improving the product reliability and quality during the product redesigning phase. The exponential related-based function is used for the computation of the separation measures from the intuitionistic fuzzy positive ideal solution (IFPIS) and intuitionistic fuzzy negative ideal solution (IFNIS) and for ranking the alternatives. The new exponential related-based function not only considers the deviation between the memberships with the non-membership degrees but also considers the hesitancy degree of the intuitionistic fuzzy set (IFS), unlike the existing matrices methods and functions that only considers the deviation between the memberships with non-membership degrees in the IFS. The weight of the evaluating criteria in this study is determined by using the intuitionistic fuzzy entropy originally proposed by Ye (2010a, b).

TOPSIS which is an abbreviation of Technique for Order Preference by Similarity to the Ideal Solution was originally proposed by Hwang and Yoon (1981) and has remained one of the most widely used MCDM methods with so many papers published on its applications (Bulgurcu 2012; Jadidi et al. 2008; Pakpour et al. 2013; Soufi et al. 2015; Yang and Wu 2008; Ghazanfari et al. 2014; Zhu et al. 2012; Chou et al. 2012).

The choice of using intuitionistic fuzzy set in this study is based on the fact that, it is more capable than the traditional fuzzy sets at handling vagueness and uncertain information in practice (Datta et al. 2013; Aikhuele and Turan 2016). Also, introducing the Fuzzy TOPSIS model in an intuitionistic fuzzy environment by using a modified exponential score function based separation method provides a whole new approach to solving multi-criteria decision-making problem. The intuitionistic fuzzy set (IFS) was introduced by Atanassov (1986), unlike the traditional fuzzy set theory, the IFS theory is characterized by a membership function and a non-membership function. The benefits of its applications have been addressed by Xu and Liao (2015), and Xu et al. (2013).

The rest of this paper is organized as follows. In “Preliminaries” section, the concepts of intuitionistic fuzzy set (IFS) theory, the exponentially related function, and the intuitionistic fuzzy entropy is presented. The intuitionistic fuzzy TOPSIS algorithm is introduced in “Algorithm of the intuitionistic fuzzy TOPSIS based on ER function (IF-TOPSIS_EF_) and the entropy weight” section. In “Illustrative examples” section a numerical case is presented to illustrate the proposed methodology. The proposed approach is compared with existing approaches in “Comparison and discussion” section while the conclusion is presented in “Conclusion” section.

## Preliminaries

In this section, the fundamental definitions and concepts of IFS theory as described by Pérez-Domínguez et al. (2015) and Despic and Simonovic (2000) is presented, also, the exponentially related function is introduced as well as the intuitionistic entropy method.

### Intuitionistic fuzzy set

**Definition 1** Consider a fuzzy set *A* in *X* = {*x*} which is given by $A = \{ \left\langle {x, \mu_{A} \left( x \right) } \right\rangle |x \in X\}$. Where $\mu_{A} :X \to \left[ {0,1} \right]$ is the membership function of the fuzzy set *A*; *μ*_*A*_(*x*) ∊ [0,1] is the membership of *x* ∊ *X* in *A.* Since IFS is characterized by two functions which expresses the degree of membership and non-membership of an element *x* to the set *A,* then an IFS *A* in *X* = {*x*} is defined as $A = \{ \left\langle {x,\mu_{A} \left( x \right), v_{A} (x)} \right\rangle | x \in X\}$, where $\mu_{A} :X \to \left[ {0,1} \right]$ and $v_{A} :X \to \left[ {0,1} \right]$ they are defined in a way that $0 \le \mu_{A} \left( x \right) + v_{A} \left( x \right) \le 1,\;\forall x \in X$.

The numbers $\mu_{A} \left( x \right) \,{\text{and}}\, v_{A} \left( x \right)$ denotes the degree of membership and degree of non-membership of element $x \in \left[ {0,1} \right]$ to the set *A* respectively. Also, the number $\pi_{A} \left( x \right) = 1 - \left( {\mu_{A} \left( x \right) + v_{A} \left( x \right)} \right)$ which is called the intuitionistic index of *x* in *A* is referred to as a measure of the degree of hesitancy of element *x* ∊ [0,1] in set *A*. It should be noted that 0 ≤ *π*_*A*_ (*x*) ≤ 1 for each $x \in X$.

**Definition 2** If the IFS *A* in *X* = {*x*} is defined fully in the form $A = \{ \left\langle {x,\mu_{A} \left( x \right), v_{A} \left( x \right), \pi_{A} \left( x \right) } \right\rangle |x \in X\}$, where $\mu_{A} :X \to \left[ {0,1} \right]$, $v_{A} :X \to \left[ {0,1} \right]$ and $\pi_{A} :X \to \left[ {0,1} \right]$. The different relations and operations for the IFS are shown in Eqs. (1–4).

1 $$A.B = \left\{ \left\langle {x,\mu_{A} \left( x \right). \mu_{B} \left( x \right), v_{A} \left( x \right) + v_{B} \left( x \right) - v_{A} \left( x \right). v_{B} \left( x \right)} \right\rangle |x \in X \right\}$$

2 $$A + B = \left\{ \left\langle {x,\mu_{A} \left( x \right) + \mu_{B} \left( x \right) - \mu_{A} \left( x \right).\mu_{B} \left( x \right),v_{A} \left( x \right). v_{B} \left( x \right) } \right\rangle |x \in X \right\}$$

3 $$\lambda A = \left\{ {\left\langle {x,1 - (1 - \mu _{A} \left( x \right))^{\lambda } ,(v_{A} \left( x \right))^{\lambda } } \right\rangle |x \in X} \right\},\quad \lambda > 0.$$

4 $$A^{\lambda } = \left\{ \left\langle {x,(\mu_{A} \left( x \right))^{\lambda } ,1 - (1 - v_{A} \left( x \right))^{\lambda } } \right\rangle |x \in X \right \} , \quad \lambda > 0$$

**Definition 3** Let $A = \left( {\mu_{j} ,v_{j} } \right), (j = 1,2,3, \ldots , n)$ be a collection of interval-valued intuitionistic fuzzy numbers and $w = (w_{1} ,w_{2} , w_{3} , \ldots ,w_{n} )^{T}$ be the weight vector of $d_{k} \left( {k = 1,2,3, \ldots , n} \right),\;w_{j} \in \left[ {0, 1} \right]$ and $\sum\nolimits_{j = 1}^{n} {w_{j} = 1}$, associated with the intuitionistic fuzzy weighted averaging (IFWA) operator (Xu 2007a). The IFWA operator is defined as;

5 $$IFWA \left( {d_{1} d_{2} d_{3} , \ldots ,d_{n} } \right) = \mathop \sum \limits_{j = 1}^{n} w_{j} d_{j} = \left(1 - \mathop \prod \limits_{j = 1}^{n} (1 - \mu_{j} )^{{w_{j} }} , \mathop \prod \limits_{j = 1}^{n} v_{j}\right)$$

In the following will make comparisons between two IFS, by introducing some metric methods by following the score function and accuracy functions.

**Definition 4** Let $A = (\mu , v)$ be an intuitionistic fuzzy number, a score function *S* of an intuitionistic fuzzy value can be represented as follow (Chen and Tan 1994; Xu 2007b);

6 $$S\left( A \right) = (\mu - v),$$

where $$S\left( A \right) \in \left[ { - 1, + 1} \right].$$

**Definition 5** Let $A = (\mu , v)$ be an intuitionistic fuzzy number, an accuracy function *H* of an intuitionistic fuzzy value can be represented as follow (Hong and Choi 2000);

7 $$H\left( A \right) = (\mu + v),$$

where $H(A) \in [0, 1]$, the larger the value of *H(A)* the more the degree of accuracy of the intuitionistic fuzzy value *A.*

**Definition 6** Let $A = (\mu , v)$ be the intuitionistic fuzzy number, according to Wu (2015) the exponential score function *S*_*e*_ of the intuitionistic fuzzy number can be represented as;

8 $$S_{e} \left( A \right) = e^{{\left( {\mu - v} \right)}}$$

where $$S(A) \in [1/e, e]$$

### The exponential related function (ER)

Considering the matrices methods reviewed above, some few drawbacks have been reported in the literature. According to Wu (2015), the results obtained using the methods are not consistent in all cases also they often produce negative priority vector in their applications. Although the exponential score function proposed by Wu (2015) appear to address these drawbacks, however, the exponential score function is design for pairwise comparison and for determining priority weight. In this paper, the exponential score function has been modified to be used for more multi-criteria analysis. The new exponential related function *ER,* not only considers the deviation between the membership degrees with the non-membership degrees but also considers the hesitancy degree of the IFS.

**Definition 7** Let $A = (\mu , v)$ be the intuitionistic fuzzy number, where *π* is the hesitancy degree of the IFS. The new exponential related function *ER* of the intuitionistic fuzzy number can be defined as;

9 $$ER\left( A \right) = \frac{{e^{{\left( {1 - \left( {|\mu - v|} \right)*\left( {1 - \pi } \right)} \right)}} }}{6}$$

The exponential related function *ER* can be rewritten as

10 $$ER\left( A \right) = \frac{{e^{{\left( {1 - (|\mu^{2} - v^{2} |)} \right)}} }}{6}$$

where $$\pi = 1 - \mu - v$$. $$ER\left( A \right) \in \left[ {0, 1} \right]$$.

*Proof* We can prove Definition 6 by mathematical induction and the similar proof method by comparing two IFNs. Next, we show that the exponential related function *ER* can achieve the same ranking as the exponential score function.

#### **Theorem 1**

*Let* $\alpha_{1} = \left( {\mu_{{\alpha_{1} }} ,v_{{\alpha_{1} }} } \right)$ *and* $\alpha_{2} = \left( {\mu_{{\alpha_{2} }} ,v_{{\alpha_{2} }} } \right)$ *be two intuitionistic fuzzy number of the exponential related function ER respectively, if α*_1_ ⊃ *α*_2_ *then* $ER\left( {\alpha_{1} } \right) > ER\left( {\alpha_{2} } \right)$.

*Proof* Assume that $\alpha_{1} = \left( {\mu_{{\alpha_{1} }} ,v_{{\alpha_{1} }} } \right)$ and $\alpha_{2} = \left( {\mu_{{\alpha_{2} }} ,v_{{\alpha_{2} }} } \right)$ are two comparable alternatives with intuitionistic fuzzy numbers based on some criteria *c*_*i*_ such that *α*_1_ ⊃ *α*_2_ without loss of generality, let assume that $\alpha_{1} > \alpha_{2 } \,{\text{i}} . {\text{e}} . ,\mu_{{\alpha_{1} }} > \mu_{{\alpha_{2} }} {\text{and}}\, v_{{\alpha_{1} }} < v_{{\alpha_{2} }}$ be such that $ER\left( {\alpha_{1} } \right) > ER\left( {\alpha_{2} } \right)$.

Now, a generalized exponential related function *ER* for an arbitrary IFN $\alpha = (\mu , v)$ can be rewritten as follows;

11 $$ER\left( {\alpha_{1} } \right) = \frac{{e^{{\left( {1 - (|\mu^{2} - v^{2} |)} \right)}} }}{6} = \frac{{\mathop \sum \nolimits e^{{\left( {1 - \mu_{{\alpha_{1j} }}^{2} } \right)}} }}{6} + \mathop \sum \nolimits 1 - \frac{{e^{{\left( {1 - v_{{\alpha_{1j} }}^{2} } \right)}} }}{6} = \frac{{e^{{\left( {1 - \mu_{{\alpha_{11} }}^{2} } \right)}} }}{6} + \frac{{e^{{\left( {1 - \mu_{{\alpha_{12} }}^{2} } \right)}} }}{6} + 1 - \frac{{e^{{\left( {1 - v_{{\alpha_{11} }}^{2} } \right)}} }}{6} + 1 - \frac{{e^{{\left( {1 - v_{{\alpha_{12} }}^{2} } \right)}} }}{6}$$

12 $$ER\left( {\alpha_{2} } \right) = \frac{{e^{{\left( {1 - (|\mu^{2} - v^{2} |)} \right)}} }}{6} = \frac{{\mathop \sum \nolimits e^{{\left( {1 - \mu_{{\alpha_{2j} }}^{2} } \right)}} }}{6} + \mathop \sum \nolimits 1 - \frac{{e^{{\left( {1 - v_{{\alpha_{2j} }}^{2} } \right)}} }}{6} = \frac{{e^{{\left( {1 - \mu_{{\alpha_{21} }}^{2} } \right)}} }}{6} + \frac{{e^{{\left( {1 - \mu_{{\alpha_{22} }}^{2} } \right)}} }}{6} + 1 - \frac{{e^{{\left( {1 - v_{{\alpha_{21} }}^{2} } \right)}} }}{6} + 1 - \frac{{e^{{\left( {1 - v_{{\alpha_{22} }}^{2} } \right)}} }}{6}$$

From Eqs. (11) and (12) *ER*(*α*_1_) − *ER*(*α*_2_) is positive

If $\alpha_{1} - \alpha_{2}$ we have;

$$= \left( {\frac{{e^{{\left( {1 - \mu_{{\alpha_{11} }}^{2} } \right)}} }}{6} + \frac{{e^{{\left( {1 - v_{{\alpha_{11} }}^{2} } \right)}} }}{6} + \frac{{e^{{\left( {1 - \mu_{{\alpha_{12} }}^{2} } \right)}} }}{6} + \frac{{e^{{\left( {1 - v_{{\alpha_{12} }}^{2} } \right)}} }}{6}} \right) - \left( {\frac{{e^{{\left( {1 - \mu_{{\alpha_{11} }}^{2} } \right)}} }}{6} + \frac{{e^{{\left( {1 - v_{{\alpha_{11} }}^{2} } \right)}} }}{6} + \frac{{e^{{\left( {1 - \mu_{{\alpha_{12} }}^{2} } \right)}} }}{6} + \frac{{e^{{\left( {1 - v_{{\alpha_{12} }}^{2} } \right)}} }}{6}} \right)$$

Is a positive number, which shows $\alpha_{1}$ is better than $\alpha_{2}$.

### The intuitionistic fuzzy entropy

Following the operations of the IFS, let us consider an intuitionistic fuzzy set *A* in the universe of discourse $X = \{ x_{1} ,x_{2 } ,x_{3 } , \ldots ,x_{n } \}$. The intuitionistic fuzzy set *A* is transformed into a fuzzy set to structure an entropy measure of the intuitionistic fuzzy set by means of $\mu_{\bar{A}} \left( {x_{i} } \right) = (\mu_{A} \left( {x_{i} } \right) + 1 - v_{A} \left( {x_{i} } \right))/2.$ Based on the definition of fuzzy information entropy Ye (2010a, b) proposes the intuitionistic fuzzy entropy as follows;

13 $$E\left( A \right) = \frac{1}{n}\sum\limits_{i = 1}^{n} {\left\{ {\left\{ {Sin\frac{{\pi *\left[ {1 + \mu_{A} \left( {x_{i} } \right) - v_{A} \left( {x_{i} } \right)} \right]}}{4} + Sin \frac{{\pi *\left[ {1 - \mu_{A} \left( {x_{i} } \right) + v_{A} \left( {x_{i} } \right)} \right]}}{4} - 1} \right\}*\frac{1}{\sqrt 2 - 1}} \right\}}$$

When the criteria weights are completely unknown, we can use the intuitionistic fuzzy entropy to determine the weights. The criteria weight is given as;

14 $$W_{j} = \frac{{1 - H_{j} }}{{n - \sum\nolimits_{j = 0}^{n} {H_{j} } }}$$

where $W_{j} \in \left[ {0, 1} \right]$, $\sum\nolimits_{j = 1}^{n} {W_{j} = 1}$, $H_{j} = \frac{1}{m}E\left( {A_{j} } \right)$ and 0 ≤ *H*_*j*_ ≤ 1 for $(j = 1, 2, 3, \ldots , n)$.

## Algorithm of the intuitionistic fuzzy TOPSIS based on ER function (IF-TOPSIS_EF_) and the entropy weight

In this section, we present the algorithm of the IF-TOPSIS_EF_ and the intuitionistic entropy weight to solve MCDM problems in which the preference information provided by DMs are expressed as intuitionistic fuzzy matrices and the matrices elements characterized by IFS value. The exponential-related function proposed herein is used for the calculation of the separation measures of each alternative from the intuitionistic fuzzy positive and negative ideal solutions (IFPIS and IFNIS) to determine the relative closeness coefficients.

Let consider a MCDM problem where a set of alternatives $A = \{ A_{1} ,A_{2} ,A_{3} , \ldots ,A_{m} \}$, are assessed with respect to the criteria denoted by $C = \{ C_{1} ,C_{2} ,C_{3} , \ldots ,C_{m} \}$. The characteristics of the alternative *A*_*i*_ with respect to a criterion *C*_*j*_ are defined first with linguistic variable and then converted to an IFS value *x*_*ij*_ = (*μ*_*ij*_, *v*_*ij*_, *π*_*ij*_) or *x*_*ij*_ = (*μ*_*ij*_, *v*_*ij*_) $(i = 1,2, \ldots ,m, \;j = 1,2, \ldots ,n)$, which represents the membership, non-membership and hesitancy degree of the alternative *A*_*i*_ ∈ *A* with respect to the criterion *C*_*j*_ ∈ *C* for the intuitionistic fuzzy concept.

The algorithm of the IF-TOPSIS_EF_ and the intuitionistic entropy weight are given in following steps;

Step 1: Set up a group of decision makers (DMs) and aggregate their evaluations; Once the DMs has given their judgment using linguistic variables, the weight vector $\lambda = \left( {\lambda_{ 1} , \lambda_{ 2} , \lambda_{ 3} , \ldots , \lambda_{l} } \right)^{T}$ is used to aggregate all DMs individual assessment matrices $DM^{k} \left( {k = 1,2,3, \ldots , l} \right)$ into the group assessment matrix (i.e. intuitionistic fuzzy decision matrix) $DM_{mxn} (x_{ij} )$ we have;

15 $$A_{{nxm}} (a_{{ij}} ) = {\text{ }}\left[ {\begin{array}{*{20}c} {(\mu _{{11}} ,v_{{11}} )} & {(\mu _{{12}} ,v_{{12}} )\,} & \ldots & {(\mu _{{1n}} ,v_{{1n}} )} \\ {(\mu _{{21}} ,v_{{21}} )} & {(\mu _{{22}} ,v_{{22}} )} & \cdots & {(\mu _{{2n}} ,v_{{2n}} )} \\ \vdots & \vdots & \ddots & \vdots \\ \vdots & \vdots & \ddots & \vdots \\ {(\mu _{{m1}} ,v_{{m1}} )} & {(\mu _{{m2}} ,v_{{m2}} )} & \cdots & {(\mu _{{mn}} ,v_{{mn}} )} \\ \end{array} } \right]$$

where $$\mu_{ij} = \left(1 - \mathop \prod \limits_{j = 1}^{n} (1 - \mu_{j})^{{w_{j} }} \right) , v_{ij} = \mathop \prod \limits_{j = 1}^{n} v_{j}$$

Step 2: Using the exponential related function *ER* (i.e. either use Eq. (9) for the three grade; *x*_*ij*_ = (*μ*_*ij*_, *v*_*ij*_, *π*_*ij*_) or Eq. (10) for membership and non-membership degrees $x_{ij} = \left( {\mu_{ij} ,v_{ij} } \right)$ convert the intuitionistic fuzzy decision matrix *DM*_*mxn*_(*x*_*ij*_) to form the exponential related matrix $ERM_{mxn} \,\left( {ER_{ij } \left( {a_{ij } } \right)} \right)$ as shown;

16 $$ERM_{{mxn}} \left( {E_{{ij}} \left( {a_{{ij}} } \right)} \right) = {\text{ }}\left[ {\begin{array}{*{20}c} {ER_{{11}} (x_{{11}} )} & {ER_{{12}} (x_{{12}} )} & \ldots & {ER_{{1n}} (x_{{1n}} )} \\ {ER_{{22}} (x_{{22}} )} & {ER_{{22}} (x_{{22}} )} & { \cdots {\text{ }}} & {ER_{{2n}} (x_{{2n}} )} \\ \vdots & \vdots & \ddots & \vdots \\ \vdots & \vdots & \ddots & \vdots \\ {ER_{{m1}} (x_{{m1}} )} & {ER_{{m2}} (x_{{m2}} )} & \cdots & {ER_{{mn}} (x_{{mn}} )} \\ \end{array} } \right]$$

Step 3: Using the intuitionistic entropy weight method as described in “The intuitionistic fuzzy entropy” section, determine the criteria weight.

Step 4: Define the IFPIS $A^{ + } = \left( {\mu_{j} ,v_{j} } \right)$ and IFNIS $A^{ - } = \left( {\mu_{j} ,v_{j} } \right)$; for the exponential related function-based matrix;

17 $$A^{ + } = \left\{ {\left\langle {C_{j} , [1, 1] \left| {C_{j} \in C} \right.} \right\rangle } \right\},\quad j = 1,2,3, \ldots ,n,$$

18 $$A^{ - } = \left\{ {\left\langle {C_{j} , \left[ {0,0} \right] \left| {C_{j} \in C} \right.} \right\rangle } \right\},\quad j = 1,2,3, \ldots ,n.$$

Step 5: Compute the exponential related *ER* function-based separation measures in intuitionistic fuzzy environment $d_{i}^{ + } (A^{ + } , A_{i} )$ and $d_{i}^{ - } (A^{ - } , A_{i} )$ for each alternative for the IFPIS and IFNIS.

19 $$d_{i}^{ + } (A^{ + },\,A_{i} ) = \sqrt {\mathop \sum \limits_{i = 1}^{n} \left[ {w_{j} \left(1 - \left( { ERM_{nxm} (a_{ij} )} \right)\right)} \right]^{2} }$$

Similarly,

20 $$d_{i}^{ - } (A^{ - } , A_{i} ) = \sqrt {\mathop \sum \limits_{i = 1}^{n} \left[ {w_{j} \left( { ERM_{nxm} (a_{ij} )} \right)} \right]^{2} }$$

where *w*_*j*_ is the weight of the criteria as described above.

Step 6: Compute the relative closeness coefficient, (*CC*_*i*_), which is defined to rank all possible alternatives with respect to the positive ideal solution *A* ^+^. The general formula is given as;

21 $$CC_{i} = \frac{{d_{i}^{ - } (A^{ - } , A_{i} ) }}{{d_{i}^{ - } (A^{ - } , A_{i} ) + d_{i}^{ + } (A^{ + } , A_{i} )}}$$

where $CC_{i} (i = 1,2, \ldots n)$ is the relative closeness coefficient of $A_{i}$ with respect to the positive ideal solution *A* ^+^ and $0 \le CC_{i} \le 1.$ The alternatives are ranked in the descending order. However, it is important to note here that since risk or failure is a negative concept, the lowest value is ranked as the highest.

## Illustrative examples

In this section, we demonstrate the computational process of the IF-TOPSIS_EF_ and the intuitionistic entropy weight model for detecting failures in product components. The result from the evaluation is hoped to provide the designer(s) with adequate information on the reliability of the product component and to guide them on designing for reliability. Also to assist the product development team in pinpointing the exact component to adjust, replace or recommended for a redesign. To ensure the effectiveness of the proposed approach, some few practical examples have been presented in this study.

**Case 1** Let us consider a multi-criteria decision-making problem originally presented by Ye (2010a) to make a new example for failure detection.

A design company wants to identify to-be-improved product components in a complex product and system in which the components are interdependent on each other. Four operational components $A_{i} \left( {i = 1,2, \ldots 4} \right)$ have been identified. However to ensure the reliability of the components as an important part of it usage data, the failure data accumulated during the usage period are evaluated to guide redesign procedure. The components are evaluated by three experts with equal expertise. The failure modes of each component are evaluated with respect to three criteria; Severity, occurrence and detection and the data are aggregated to form the intuitionistic fuzzy decision matrix $DM_{4x3} (a_{ij} )$ as shown Table 1 below.

**Table 1:** Intuitionistic fuzzy decision matrix for the product components

Product components	Severity	Occurrence	Detection
PC1	(0.45, 0.35)	(0.50, 0.30)	(0.20, 0.55)
PC2	(0.65, 0.25)	(0.65, 0.25)	(0.55, 0.15)
PC3	(0.45, 0.35)	(0.55, 0.35)	(0.55, 0.20)
PC4	(0.75, 0.15)	(0.65, 0.20)	(0.35, 0.15)

Using the exponential-related function in Eq. (10), the intuitionistic fuzzy decision matrix $DM_{4x3} (a_{ij} )$ is converted to form the exponential related matrix $ERM_{4x3} \left( {ER_{ij } \left( {a_{ij } } \right)} \right)$ as show in the Table 2. Also, by following the implementation procedure for the intuitionistic fuzzy entropy, the weights of the criteria are determined, the weight results for the three criteria are given as W = {0.313, 0.377, 0.311} respectively.

**Table 2:** The exponentially related matrix, the distance measures and the relative closeness coefficients of the failure modes for the four components

Product components	Severity ER	Occurrence ER	Detection ER	$d_{i}^{ + }$	$d_{i}^{ - }$	*CC*_*i*_	Ranking
PM1	0.418	0.386	0.348	0.357	0.224	0.385	4
PM2	0.316	0.316	0.342	0.393	0.188	0.324	1
PM3	0.418	0.378	0.348	0.359	0.222	0.382	3
PM4	0.264	0.309	0.410	0.393	0.191	0.327	2

Finally, by using Eqs. (16) and (17), the exponential related function-based separation measures $d_{i}^{ + } (A^{ + } , A_{i} )$ and $d_{i}^{ - } (A^{ - } , A_{i} )$ $\left( {i = 1, 2, \ldots , 1 6} \right)$ is calculated, also the relative closeness coefficient $CC_{i} , (i = 1,2, \ldots ,16)$ to the ideal solution is calculated using Eq. (18). The results are shown in Table 2.

The ranking of the failure modes for the four components as shown in Table 2 is in agreement with the result obtained in (Ye 2010a).

**Case 2** Let us consider a practical failure detection problem originally presented by Chang and Wen (2010) and adopted by Liu et al. (2015). In this case, the original problem has been slightly modified to make a new example.

A design company wants to identify to-be-improved product components in a complex product and system in which the components are interdependent on each other. Sixteen operational components $A_{i} \left( {i = 1,2, \ldots 16} \right)$ have been identified through traditional QFD method. However to ensure the reliability of the components as an important part of it usage data, the failure data accumulated during the usage period are evaluated to guide redesign procedure. The components are evaluated by three experts with equal expertise. The failure modes of each component are evaluated with respect to three criteria; Severity, occurrence and detection and the data are aggregated to form the intuitionistic fuzzy decision matrix $DM_{nxm} (a_{ij} )\varvec{ }$ as shown Table 3 below.

**Table 3:** Intuitionistic fuzzy decision matrix for the product components

Product components	Severity	Occurrence	Detection
PM1	(0.337, 0.543)	(0.566, 0.290)	(0.386, 0.516)
PM2	(0.380, 0.514)	(0.467, 0.467)	(0.418, 0.495)
PM3	(0.421, 0.490)	(0.645, 0.204)	(0.124, 0.739)
PM4	(0.519, 0.383)	(0.472, 0.464)	(0.373, 0.519)
PM5	(0.329, 0.548)	(0.540, 0.344)	(0.244, 0.636)
PM6	(0.235, 0.626)	(0.540, 0.344)	(0.277, 0.598)
PM7	(0.129, 0.733)	(0.623, 0.218)	(0.148, 0.715)
PM8	(0.171, 0.678)	(1.000, 0.000)	(0.240, 0.629)
PM9	(0.472, 0.464)	(0.495, 0.413)	(0.161, 0.696)
PM10	(0.579, 0.268)	(0.556, 0.312)	(0.519, 0.383)
PM11	(0.279, 0.587)	(0.553, 0.335)	(0.337, 0.543)
PM12	(0.400, 0.500)	(0.606, 0.256)	(0.358, 0.528)
PM13	(0.287, 0.582)	(0.636, 0.208)	(0.532, 0.377)
PM14	(0.306, 0.563)	(0.524, 0.371)	(0.232, 0.635)
PM15	(0.421, 0.490)	(0.522, 0.400)	(0.051, 0.822)
PM16	(0.376, 0.520)	(0.447, 0.477)	(0.358, 0.528)

Using the exponential-related function in Eq. (10), the intuitionistic fuzzy decision matrix $DM_{16x3} (a_{ij} )\varvec{ }$ is converted to form the exponential related matrix $ERM_{16x3} \left( {ER_{ij } \left( {a_{ij } } \right)} \right)$ as show in the Table 4. Also, by following the implementation procedure for the intuitionistic fuzzy entropy, the weights of the criteria are determined, the weight results for the three criteria are given as W = {0.232, 0.349, 0.419} respectively.

**Table 4:** The exponentially related matrix, the distance measures and the relative closeness coefficients of the failure modes for the product components

Product components	Severity ER	Occurrence ER	Detection ER	$d_{i}^{ + }$	$d_{i}^{ - }$	*CC*_*i*_	Ranking
PM1	0.543	0.358	0.509	0.322	0.278	0.463	4
PM2	0.511	0.453	0.486	0.309	0.284	0.478	6
PM3	0.482	0.312	0.770	0.285	0.358	0.557	13
PM4	0.401	0.450	0.516	0.312	0.283	0.476	5
PM5	0.549	0.381	0.640	0.284	0.325	0.534	11
PM6	0.634	0.381	0.600	0.286	0.320	0.528	10
PM7	0.763	0.322	0.739	0.266	0.374	0.584	15
PM8	0.697	0.167	0.635	0.336	0.317	0.485	7
PM9	0.450	0.421	0.717	0.267	0.350	0.567	14
PM10	0.348	0.367	0.401	0.367	0.226	0.381	1
PM11	0.592	0.373	0.543	0.306	0.296	0.492	8
PM12	0.496	0.335	0.527	0.327	0.275	0.457	3
PM13	0.585	0.316	0.394	0.362	0.240	0.399	2
PM14	0.566	0.395	0.643	0.278	0.330	0.543	12
PM15	0.482	0.405	0.888	0.244	0.413	0.628	16
PM16	0.515	0.466	0.527	0.294	0.299	0.504	9

Finally, by using Eqs. (16) and (17), the exponential related function-based separation measures $d_{i}^{ + } (A^{ + } , A_{i} )$ and $d_{i}^{ - } (A^{ - } , A_{i} )$ $\left( {i = 1, 2, \ldots , 1 6} \right)$ is calculated, also the relative closeness coefficient $CC_{i} , (i = 1,2, \ldots ,16)$ to the ideal solution is calculated using Eq. (18). The results are shown in Table 4.

## Comparison and discussion

To further demonstrate the effectiveness of our proposed model for failure detection, we compare the results of the example in case 4 by analyzing the case with some similar computational approaches including the fuzzy TOPSIS model by Braglia et al. (2003), the integrated weight-based fuzzy TOPSIS (IWF-TOPSIS) by Song et al. (2013), the intuitionistic fuzzy hybrid TOPSIS (IFH-TOPSIS) approach by Liu et al. (2015) and the risk priority number (RPN) method. The final ranking results are shown in Table 5.

**Table 5:** Ranking of failure in a complex product using different approaches

Product components	Proposed model	Fuzzy TOPSIS model	IWF-TOPSIS	IFH-TOPSIS	RPN method
PM1	4	9	10	7	6
PM2	6	13	8	9	10
PM3	13	4	5	5	9
PM4	5	6	2	6	3
PM5	11	11	11	11	14
PM6	10	15	14	15	10
PM7	15	16	16	16	15
PM8	7	2	15	4	13
PM9	14	7	3	8	8
PM10	1	1	1	1	1
PM11	8	10	13	10	6
PM12	3	3	4	3	4
PM13	2	5	7	2	2
PM14	12	14	12	14	10
PM15	16	10	9	13	16
PM16	9	15	6	12	5

The rankings of the different approaches show that they are almost in agreement with our proposed approach. The result shows that except for PMs 6, 7, 10, 13 and 15, the failure modes for other PM obtained by our proposed method are different from those obtained using the conventional RPN method. However, the difference is as a result of the limitation in FMEA method as mentioned in “Background” section.

The advantages of the proposed method in this study over the conventional FMEA methods (RPN) can be summarized to include;

- The ability of the new method to reflected and modeled the fuzziness and hesitation of the experts’ subjective assessments of the failure modes with the application of the exponential-related function.
- The method considers not only the deviation between the membership and non-membership degrees as obtainable in other intuitionistic methods but considers also the hesitancy degree of the IFS.
- The results from the proposed method for failure detection are more objective and reliable because the criteria weights were determined using an objective weight approach.
- The implementation procedures of the proposed model and approach are easy and straightforward as compared to the other Multi-criteria decision-making methods and TOPSIS approaches compared in this study.

## Conclusion

The main goal of any product redesigning exercise is to create new products and systems that meet the customer requirements as well as the product reliability. In identifying product component(s) to be redesigned, the customer/user requirements which are mainly considered and achieved through customer surveys using the quality function deployment (QFD) tool, often fail to guarantee or cover aspects of the product reliability. Even when they do, there are always many misunderstandings.

To improve the product reliability and quality during the product redesigning phase and to create that novel product(s) for the customers, the failure or potential failure information of the existing product, and its component(s) should ordinarily be identified and analyzed. In this study, we have presented an intuitionistic fuzzy TOPSIS model which is based on an exponential-related function for building appropriate design knowledge about the to-be-improved or redesigned product component by analyzing the historical failure information about the product with the view to prioritizing the failure modes and to provide the designer with relevant information to be used in improving the product reliability and quality during the product designing phase. The exponential related-based function has been used for the computation of the separation measures from the intuitionistic fuzzy positive ideal solution (IFPIS) and intuitionistic fuzzy negative ideal solution (IFNIS) and for ranking the alternatives. The new exponential related-based function not only considers the deviation between the memberships and the non-membership degrees but also considers the hesitancy degree of the intuitionistic fuzzy set (IFS). The weight of the evaluating criteria in this study has been determined using the intuitionistic fuzzy entropy originally proposed by Ye (2010a, b).

To demonstrate the effectiveness of the proposed approach for failure detection, two practical case studies have been presented and evaluated using the proposed approach. Also, the results from the different case study have been compared with some similar computational approaches such as; the conventional Fuzzy TOPSIS model, the integrated weight-based fuzzy TOPSIS (IWF-TOPSIS) model, the intuitionistic fuzzy hybrid TOPSIS (IFH-TOPSIS) approach and finally with the conventional risk priority number (RPN) method.

Finally, we can conclude that the new approach proposed in this study, provides a better alternative method for failure identification and analysis as it allows for the fuzziness and hesitation of the experts’ subjective assessments to be reflected and modeled in the evaluation. Also, in applying the proposed model, the particular fault area, and the failed component can easily be identified. In the future, it is recommended that the proposed approach should be applied in solving other multi-criteria decision-making problems with special reference to the two ranking order for the two exponential related functions.

## Abbreviations

TOPSIS: Technique for Order Preference by Similarity to the Ideal Solution
FMEA: failure mode and effect analysis
QFD: quality function deployment
IFPIS: intuitionistic fuzzy positive ideal solution
IFNIS: intuitionistic fuzzy negative ideal solution
IFS: intuitionistic fuzzy set
IFWA: intuitionistic fuzzy weighted averaging
ER: exponential related function
